# A Shared Neural Substrate for Mentalizing and the Affective Component of Sentence Comprehension

**DOI:** 10.1371/journal.pone.0054400

**Published:** 2013-01-16

**Authors:** Pierre-Yves Hervé, Annick Razafimandimby, Gaël Jobard, Nathalie Tzourio-Mazoyer

**Affiliations:** 1 Univ. Bordeaux, Groupe d’Imagerie Neurofonctionnelle, UMR 5296, Bordeaux, Aquitaine, France; 2 Centre National de la Recherche Scientifique, Groupe d’Imagerie Neurofonctionnelle, UMR 5296, Bordeaux, Aquitaine, France; 3 Commissariat à l’ Énergie Atomique, Groupe d’Imagerie Neurofonctionnelle, UMR 5296, Bordeaux, Aquitaine, France; 4 Imagerie et Stratégies Thérapeutiques de la Schizophrénie, Unité Mixte de Recherche 6301, CNRS, CEA, Univ. Caen Basse-Normandie, Caen, Basse-Normandie, France; University of Montreal, Canada

## Abstract

Using event-related fMRI in a sample of 42 healthy participants, we compared the cerebral activity maps obtained when classifying spoken sentences based on the mental content of the main character (belief, deception or empathy) or on the emotional tonality of the sentence (happiness, anger or sadness). To control for the effects of different syntactic constructions (such as embedded clauses in belief sentences), we subtracted from each map the BOLD activations obtained during plausibility judgments on structurally matching sentences, devoid of emotions or ToM. The obtained theory of mind (ToM) and emotional speech comprehension networks overlapped in the bilateral temporo-parietal junction, posterior cingulate cortex, right anterior temporal lobe, dorsomedial prefrontal cortex and in the left inferior frontal sulcus. These regions form a ToM network, which contributes to the emotional component of spoken sentence comprehension. Compared with the ToM task, in which the sentences were enounced on a neutral tone, the emotional sentence classification task, in which the sentences were play-acted, was associated with a greater activity in the bilateral superior temporal sulcus, in line with the presence of emotional prosody. Besides, the ventromedial prefrontal cortex was more active during emotional than ToM sentence processing. This region may link mental state representations with verbal and prosodic emotional cues. Compared with emotional sentence classification, ToM was associated with greater activity in the caudate nucleus, paracingulate cortex, and superior frontal and parietal regions, in line with behavioral data showing that ToM sentence comprehension was a more demanding task.

## Introduction

Humans are able to build representations of the contents of the mind of others, such as their beliefs, desires or intentions, and this enables them to understand, predict or act on the behavior of others [1]. These complex representations of the cognitive or emotional mental states of others may include what they know, or don’t know of a shared situation, as well as their long-term goals or salient psychological traits. This capacity is usually referred to as theory-of-mind (ToM), intentional stance, cognitive empathy, folk psychology or mentalizing, and has been associated with the activity of a number of cortical areas, including the dorsomedial prefrontal cortex (dmPFC), ventromedial prefrontal cortex (vmPFC), temporo-parietal junction (TPJ), posterior cingulate cortex (pCC) and anterior temporal lobe (aTL) [2].

ToM is aimed at identifying the reasons for the recognized actions or emotions of others, which is different from their recognition. As explained by Sabbagh [3]: “*in order to correctly infer that someone is sad because she got a poor mark on an exam one needs to detect sadness from the observable information, know that she received a poor mark, and perhaps know that she had wanted to do well*”.

These distinct, but interrelated emotion recognition and mentalizing processes seem to rely on different neural systems. In the particular context of emotional speech processing, Beaucousin *et al.* suggested that the left inferior frontal and right superior temporal areas are involved in the recognition of emotions respectively through emotional lexico-semantic cues and affective prosodic cues [4]. Crucially, regions strongly associated with ToM processing, namely the dorsal mPFC and left TPJ, were also recruited during the same experiment, irrespective of the presence of emotional prosody. The application of functional connectivity analyses to a second fMRI dataset further revealed that the large set of brain regions involved in emotional classification could be subdivided into two main functional networks [5]: one that gathered perisylvian language areas, and one that overlapped ToM regions [2]. Given this involvement of a distinct coherent network of putatively ToM-related regions (“Medial network”, including the pCC, left TPJ, dmPFC and vmPFC), it seemed even more likely that the emotional sentence processing entailed a form of ToM process.

The fact that the Medial network included the vmPFC was particularly interesting. The function of this region has been theorized as “*a hub that connects systems involved in episodic memory, representation of the affective qualities of sensory events, social cognition, interoceptive signals, and evolutionarily conserved affective physiological and behavioral responses*”, that “*bridges conceptual and affective processes*” [6]. Accordingly, in an earlier review of imaging studies on the neural bases of human social cognition, the ventral part of the mPFC was discussed as likely to contain a “*distinct neural substrate of emotional empathy*” [2]. The vmPFC and the orbitofrontal cortex are considered key regions for affective ToM [7]–[10], which deals with the representation of the emotional states of others. Affective ToM shows a large neural overlap with the cognitive aspects of ToM (dealing with thoughts, beliefs, intentions or desires) [7], [8], and is the particular facet of ToM that could be engaged during affective speech processing.

In order to verify that, in the same group of participants, the same brain regions that support emotional speech processing are also involved in ToM, we scanned the volunteers who had performed the emotional sentence classification tasks a second time, with a new classification task on sentences describing mental contents. Contrary to the emotional sentence classification task, where the participants were only asked to classify the sentences according to their emotional content, this task used explicit mentalizing instructions. Compared with emotional situations, the verbal description of mentalizing situations involved longer and more complex sentences, including several characters and embedded clauses (especially second-order beliefs, e.g. “he thinks that she thinks that…”). So as to avoid the confound of a different syntactic complexity between the ToM and emotional sentence classification tasks, we conceived two plausibility judgment tasks on sentences that were matched on a one-to-one basis with the ToM or emotional sentences, in terms of their number of words, verbs and clauses. These structurally matched reference sentences were devoid of ToM or emotional contents. This enabled the comparison of mentalizing and the emotional aspects of sentence comprehension, while controlling for the effects of differing syntactic constructions.

## Materials and Methods

### Ethics Statement

The local ethics board (CCPRB: Comité Consultatif de Protection des Personnes se Prêtant à la Recherche Biomédicale, Basse-Normandie) had approved the experimental protocol. The participants gave their informed, written consent, and received an allowance for their participation.

### Participants

From the 51 participants to the previous study for which the emotional speech processing data were acquired [5], we included a total of 42 participants (26 males), comprising 2 left-handers (1 male), who were available for a second fMRI experiment. This allowed the comparison of the different conditions in the same set of participants. The mean Edinburgh score of right-handers was 93.3 (standard deviation = 13.6), while it was −100.0 for the left-handers. The median age of the group was 27.5 years (mean ± sd: 30.9±8.6 years, range 18–53 years). The average level of education was 15.9 years ±3.4 years, minimum: 11 years, maximum: 20 years) corresponding to 4 years of education after the baccalaureate. Note that there was no correlation between age and level of education in this group. We have not detected any abnormality in the structural scans of any of the included participants.

### Cognitive Tasks

#### TOM and PLAUTOM tasks

In the TOM task, the participants were asked to classify 48 French sentences into 3 different categories on the basis of the mental state they attributed to the main character: belief, deception, or empathy. The complete set of sentences used in TOM and other tasks is presented as supplementary material (Materials S1).

Belief sentences could correspond to a 1^st^ order situation, when one has a conviction that is unfounded *(With his rabbit-foot in his pocket, he is sure to win the race)*, a belief based on an appearance that is different from reality (*Because of her disguise, the cafe’s landlord directed her to the men’s toilets*) or to 2^nd^ order situations involving beliefs about the intentions of another person *(His girlfriend does not talk to him about their next holidays because she thinks that he is going to leave her; After what happened between them, she does not think that he will have the audacity to meet her again)*. Deception sentences corresponded to situations where a character deliberately lies (*Arrested for running a light, the driver maintains to the policeman that she went when the light was green; Despite the smell, he assures his client that his fish is fresh)*, or dissimulates his intentions (*Anticlerical, he praises the pope with his electoral speech to attract the Catholics*). Empathy sentences corresponded to situations where one shares or takes into account another person’s feelings or emotions *(On seeing his smiling face when arriving, she feels that he shares the pleasure of this meeting; When they announce to the patient that his tumour is benign, the doctors are pleased to see the patient’s relief; To not ruin Pierre’s party, nobody told him that he sang flat)*.

The 48 TOM sentences included in the fMRI paradigm (16 sentences of each category) were selected from an initial corpus composed of one set of 26 sentences (belief) and two sets of 24 sentences (deception and empathy, 74 total). In order to select the best 48 sentences out of the initial set of 74, a group of 14 participants completed a preliminary experiment. The participants were asked to classify the set of 74 sentences into the three categories. A total of 26 sentences had to be excluded. To do so, we removed the most ambiguous sentences (eliciting less than 7 correct responses, *i.e.* correctly classified by less than 50% of the subjects) as well as those that were too easy (as shown by a response time below 500 ms together with a number of correct responses close to 14). This left an excess of 16 valid sentences, which were chosen randomly. Nine of these extra sentences (3 for each category) and their matched reference sentences were used in a training session with the TOM and PLAUTOM tasks prior to the fMRI experiment.

In PLAUTOM, participants had to evaluate whether a sentence – which had a correct syntactic construction – was plausible or not. Out of the 48 sentences, 15 (31.25%) were implausible. Note that all TOM sentences were plausible. For each PLAUTOM sentence, the same syntactic structure as the matching TOM sentence was employed, while the semantic content was altered. For instance, the implausible sentence matching the deception sentence “*Her meeting cancelled, she however tells her husband that she is going to work late tonight*” was “*The marathon over, the pain tells the runner that his muscles will be* redacted *tonight*”. By construction, the sentences used in the PLAUTOM task thus were matched on a one-to-one basis with the TOM sentences, in terms of their length, number of words, number of verbs, and number of clauses (see Table 1). Two-sample t-tests or Chi-squared tests comparing the TOM stimuli to their PLAUTOM references did not show any significant difference in terms of the duration of the stimuli (*p* = 0.38), or the number of words (*p* = 0.55), verbs (*p* = 1.0), clauses (*p* = 0.31) and adjectives (*p* = 0.66) in the sentences. On the opposite, there was a highly significant difference between these two tasks concerning the number of characters involved per sentence (*p*<0.0001, Table 1), in keeping with the social nature of the TOM stimuli.

**Table 1:** Descriptive statistics for the sentences used in the 5 classification tasks (mean ± sd).

	TOM	PLAUTOM	EMO	PLAUEMO	GRAM
Duration(sec)	4.68±0.84	4.81±1.04	2.65±0.49	3.12±0.62	2.64±0.49
N words	15.52±2.78	15.81±3.04	10.75±2.13	11.52±2.29	9.46±1.57
N verbs	3.40±1.18	3.40±1.25	2.23±0.69	2.15±0.71	2.17±0.72
N characters	2.12±0.49	0.48±0.95	1.78±0.78	0.65±0.81	1.27±0.61
N adjectives	0.71±0.65	0.83±0.75	0.65±0.70	0.42±0.61	0.31±0.47
N clauses	2.75±0.70	2.96±0.71	1.40±0.49	1.44±0.50	1.15±0.36

#### EMO, GRAM and PLAUEMO tasks

The volunteers had previously performed two different runs of emotional sentence and neutral sentences classification (EMO and GRAM tasks, see [4], [5] for details). As in the TOM protocol, the participants heard a total of 48 sentences. All EMO sentences were plausible. The participants were asked to classify the emotional message conveyed by the sentence into 3 categories (*“happy”, “angry” or “sad”*). In the GRAM task, the participant had to classify the sentences according to the subject of the sentence (*“I”, “you” or “he/she”*). Regarding the new PLAUEMO reference task, similar to PLAUTOM, the sentences matched the EMO sentences on a one-to-one basis and lacked emotional content. The volunteers had to evaluate whether the sentences were *plausible* or *implausible* (15 implausible sentences out of 48). The duration of the PLAUEMO sentences was slightly, but significantly higher than both EMO and GRAM sentences (3.12 s versus 2.65 and 2.64 s, both *p-*values <0.0015, Table 1). The average durations of EMO and GRAM sentences were not significantly different (Table 1, [5]). EMO sentences did not differ significantly from PLAUEMO sentences in terms of their total numbers of words (*p* = 0.12), verbs (*p* = 0.66), clauses (*p* = 0.68) or adjectives (*p* = 0.18), but EMO sentences contained significantly more characters (*p*<0.0001).

There were more characters in TOM sentences than in EMO sentences (*p* = 0.02), whereas the number of characters did not differ significantly between PLAUTOM and PLAUEMO (*p* = 0.27). There were more characters in GRAM than either PLAUEMO or PLAUTOM (both *p*<0.0001), and more characters in EMO than GRAM (*p* = 0.0012). As for the comparison of the long sentences (TOM and PLAUTOM) and short sentences (EMO, GRAM and PLAUEMO tasks), as expected, the number of words was significantly higher in the long-sentences paradigm (all *p*-values <0.0001, Table 1).

### fMRI Protocol

The TOM, PLAUTOM and PLAUEMO data were acquired in a second fMRI session with the same participant, several months (10.5 on average) after a first session during which the EMO and GRAM data were acquired. Each participant performed two different runs of each task while in the magnet, for a total of 10 runs over both sessions. For each task, each run included 24 sentences, and was organized following a slow event-related design (with a long interval between each stimulus so as to allow the BOLD response to go back to baseline). The different sentence categories occurred randomly, but in the same order for all participants. After the end of the sentence, the participants had to respond manually within 3 s. In order to keep the participants focused on the experiment, after each sentence classification trial, the participants performed a “beep detection task”. They heard the same two tones in a random order, separated by 2 to 8 s, and had to respond upon hearing the lower-frequency tone. For the EMO, PLAUEMO and GRAM tasks, the total event duration (sentence classification plus beep detection) was 14±2 s. For TOM and PLAUTOM, with longer sentences, the interval was 16±2 s. The pulses sent by the MRI scanner triggered the onsets of the events.

In all paradigms the presentation of the stimuli and recording of responses were done using the E-Prime 1.2 software. The auditory stimuli were delivered via MR compatible headphones (MR-CONFON Gmbh), and the manual responses were collected using an MR-compatible response-pad (Current Designs).

### Debriefing

Shortly after the scanning session, the participants completed a structured debriefing interview. The same questions, as written on an interview form, were asked to the different participants. The experimenter asked the questions and filled in the responses on the interview form. After answering general questions, participants had to report on their strategy during the TOM task. Using the form, the experimenter recorded whether or not the participant had used the following indices or strategy to classify the ToM sentences: simulation of ones’ mental state, reliance on social knowledge, analysis of the sentences’ lexical content (including the analysis of a specific grammatical category such as verbs and adjectives), analysis of the sentences’ structure, silent sentence rehearsal, mental imagery of complex scenes, analysis of prosody. The debriefing also included questions about the way the participants solved the plausibility tasks: sentence rehearsal, analysis of lexical content, attention to words situated at a particular position in the sentence, silent rehearsal of the sentences, prosody, mental imagery.

### Image Acquisition

The data were acquired on the Philips Intera Achieva 3T scanner at the GIP Cyceron (Caen, France). The anatomical scans consisted of a T1-W sagittal 3D scan (180×256×256 voxels, with a 1-mm isotropic resolution, FA = 10°, TE = 4.6 ms, TR = 20 ms, TI = 800 ms, SENSE factor = 2 in both AP and LR directions), and of a coarser T2*-W scan, with a T2-FFE sequence (128×128×70 voxels, 2 mm isotropic resolution, TE = 30 ms, TR = 3500 ms, FA = 90°, SENSE factor = 2), for cross-modal registration with the EPI-BOLD time-series. For the functional MRI, the sequence parameters were: 31 axial slices with a 64×64 matrix, 3.75 mm isotropic voxel resolution, interleaved acquisition, TE = 35 ms, TR = 2 s, FA = 80°, no parallel imaging.

### Image Processing

Image analysis was performed using the SPM5 software. The T1-weighted scans of the participants were normalized to a site-specific template (T-80TVS) matching the MNI space, using the SPM5 “segment” procedure with otherwise default parameters. So as to correct for subject motion during the fMRI runs, within each run, the EPI-BOLD scans were realigned using a rigid-body registration. The EPI-BOLD scans then were registered rigidly to the structural T2-weighted image, which was itself registered to the T1-weighted scan. The combination of all registration matrices allowed warping the EPI-BOLD functional scans to the standard space. Once in the standard space, a 6-mm FWHM Gaussian filter was applied.

### Behavioral Data Analysis

We compared the accuracy (mean number of correct responses per run, CR), the response times (RT) of correct answers, between the TOM and PLAUTOM tasks using Wilcoxon rank tests. Likewise, we compared the RTs and CRs of the PLAUEMO task with those of the previously described EMO and GRAM tasks [5]. We also evaluated the effects of sex, age and education level on RTs for each of the 5 tasks, using linear models.

On the basis of the participants’ answers to the debriefing questionnaires, we computed descriptive statistics regarding the various task-solving strategies used by the participants to complete the TOM and PLAU tasks.

### Statistical Analysis of Functional Data

#### Subject-level analyses

Regarding the functional imaging data, for the 1^st^ level (individual) analyses, we used the SPM General Linear Model. For each of the 10 runs, we used a single stimulus function, convolved with the canonical hemodynamic response function. The different types of events within each run were not separated. The event durations included the response time. The motion parameters, as estimated by the motion correction procedure, were included into the model.

#### Group level statistical parametric mapping

At the 2^nd^ level (group analysis), for the comparisons between tasks, we used a repeated-measures ANOVA design (flexible factorial design), with a Task factor with 5 levels (EMO, PLAUEMO, GRAM, TOM, PLAUTOM). We had 2 contrast images for each subject in each level (*i.e.* one image per run). A Subject factor with 42 levels accounted for the between-subject variability. So as to be able to assess the conjunction between the TOM and PLAUTOM tasks, we computed a similar model, albeit without the Subject factor. Note that tables report only clusters with more than 10 voxels in order to limit their sizes.

#### ToM versus non-ToM sentence processing

So as to highlight the brain regions involved in ToM relative to sentence comprehension, we first contrasted the TOM and PLAUTOM sentence classification task (*p*<0.05, FWE correction for multiple comparisons), masking for positive signal variations during TOM. Using the model without the Subject factor, we also computed the conjunction between the TOM and PLAUTOM sentence comprehension tasks (*p*<0.05, voxel-wise FWE correction for multiple comparisons). In this case TOM or PLAUTOM activations were measured relative to the baseline (beep detection).

#### Overlap between emotional and ToM sentence processing

To identify the regions that respond to both ToM and emotional sentence classification conditions more than to plausibility judgment tasks on sentences, we computed the conjunction between the [TOM – PLAUTOM] and [EMO – PLAUEMO] contrasts. Of note, some unspecific overlap between the two contrasts could occur for two different reasons, namely the task-related deactivations common to PLAUEMO and PLAUTOM, and the comparison of 3-choice (*belief, empathy, deception*) tasks to 2-choice tasks. In order to avoid such confounds, we masked the contrasts of interest so as to include only voxels in which the [EMO – GRAM] contrast is significant, at a voxel-wise threshold of *p<*0.0001, uncorrected for multiple comparisons. The GRAM condition is indeed devoid of emotional or ToM material, but may not induce the same deactivations as PLAUEMO and PLAUTOM, and has the same number of possible responses as TOM or EMO. We thus used the two different reference tasks for EMO to increase specificity.

#### Differences between emotional and ToM sentence processing

In order to distinguish the regions that are more involved during ToM sentence processing than during emotional sentence processing, and *vice versa*, we computed the two possible one-sided comparisons between the [TOM – PLAUTOM] and [EMO – PLAUEMO] contrasts (i.e. the two interaction contrasts). The statistical threshold was again set at *p*<0.05 with a voxel-wise FWE correction for multiple comparisons. For the [EMO – PLAUEMO] – [TOM – PLAUTOM] contrast, showing the regions more associated with emotional than with ToM sentence processing, we also masked the results inclusively by the [EMO – GRAM] contrast at *p*<0.0001 uncorrected, in keeping with the previous conjunction analysis. The reverse contrast was masked inclusively by [TOM – PLAUTOM] activations at an uncorrected threshold (*p*<0.0001 voxel-wise), so as to exclude results driven uniquely by greater activations during PLAUEMO compared with EMO.

#### ROI based analyses

So as to test the hypothesis that the “Medial network” regions identified in the previous study [5] are active during mentalizing, we extracted the contrast values for the 5 tasks in each of the 6 regions-of-interest (ROIs, radius of 4 mm). These 6 ROIs consisted of the bilateral dmPFC (3 ROIs, with MNI x y z coordinates triplets, in mm: −6 56 34, 6 54 36, 6 58 24), the vmPFC (at −2 46 −12), the pCC (at −4 50 28) and the left TPJ (at −42 60 26). We applied two-sided t-tests on the TOM, PLAUTOM, PLAUEMO and TOM – PLAUTOM contrasts (each time using the average of the two replications, with 41 degrees of freedom), with a Holm-Bonferroni stepwise correction for multiple comparisons (6 null-hypotheses), within each of the 4 contrasts. The EMO and GRAM conditions were not tested as they had been used for ROI definition and this analysis would have been circular.

We performed further analyses in the vmPFC, aimed at assessing the specific hypothesis that the *Empathy* condition of the TOM task, due to its affective component, would show a stronger response than *Belief* or *Deception.* We used 4-mm ROIs positioned over peaks of stronger activity during TOM, EMO or both in the whole brain analyses, and data from similar 1^st^ level SPM models as presented above, except with separate stimulus functions for each of the 3 conditions. We performed the two-by-two comparisons between the 3 TOM conditions using paired two-sample t-tests. No correction for multiple comparisons was applied in this exploratory analysis.

## Results

### Behavioural Data

#### ToM and PLAUTOM tasks

Descriptive statistics are presented in Table 2. For both TOM and PLAUTOM tasks, accuracy was high, and PLAUTOM was better succeeded to than TOM (Wilcoxon test on CR: *p*<0.0001). Accordingly, RTs were significantly higher during TOM as compared with PLAUTOM (*p*<0.0001).

**Table 2:** Behavioral data for the 5 tasks (mean ± SD).

	TOM	PLAUTOM	EMO	PLAUEMO	GRAM
RC(total 24)	20.33±1.84	22.65±1.06	23.60±0.53	22.56±1.26	23.57±0.52
RT (ms)	935±252	703±218	720±250	754±194	620±228

#### PLAUEMO, EMO and GRAM tasks

We observed a slightly, but significantly higher accuracy during EMO or GRAM compared with PLAUEMO (both tests: *p*<0.0001, Table 2), and significantly shorter response times for GRAM compared with either PLAUEMO (*p*<0.0001) or EMO (*p*<0.0001, as previously shown [5]). The RTs during EMO and PLAUEMO and the number of CR during EMO and GRAM did not differ significantly (*p* = 0.17 and *p* = 0.95 respectively). The participants also displayed significantly longer RTs during PLAUEMO than during PLAUTOM (*p* = 0.0056, Table 2), but their accuracy was not significantly different between these two tasks (*p* = 0.62). The RTs were significantly longer and CR numbers significantly lower during TOM compared with the PLAUEMO, EMO, or GRAM conditions (all *p*-values <0.0001).

#### Effects of age, sex and education level

There was no significant effect of age or sex on the RTs for any of the 5 tasks (EMO, age: *p* = 0.34, sex: *p* = 0.78; GRAM, age: *p* = 0.87 p = 0.27; PLAUEMO, age: *p* = 0.69, sex: *p* = 0.36; TOM, age: *p* = 0.60, sex: *p* = 0.47; PLAUTOM, age: *p* = 0.83, sex: *p* = 0.18). More years of education, however, were associated with faster responses at the TOM task (*p*<0.05). A similar but non-significant trend was observed for EMO and GRAM (*p*<0.1), but not PLAUTOM (*p* = 0.33) or PLAUEMO (*p* = 0.30).

#### Debriefing questionnaire

Regarding the strategies employed by the 42 participants during the TOM task, 81% of them reported to have relied on their experience of social interactions. Simulation of the characters perspective was reported by 57% of the participants. The verbs of the sentences were a useful cue for 83% of the participants. Adjectives were useful according to 50% of the participants. Mental imagery of complex scenes was reported by 62% of the participants. Mental imagery of a dialogue (conversation with the speaker) was reported by only 4 participants (9%), and feeling emotions by 6 participants (14%). Eight participants reportedly relied on intonation (19.5%). Mental rehearsal of the sentences was reported by 62% (26) of the participants.

As expected, in order to solve the PLAU tasks, the participants relied on the meaning of the sentences and words (95 and 90% of the participants), and paid particular attention to words located at the end of the sentence (72.5% of participants). This task entailed mental imagery of the sentences’ content in 65% of the participants (20 out of the 31 participants to whom we had asked this question), of whom 45% (9) reported that this was helpful.

### fMRI Data

#### Areas involved in sentence comprehension

**Figure 1:**
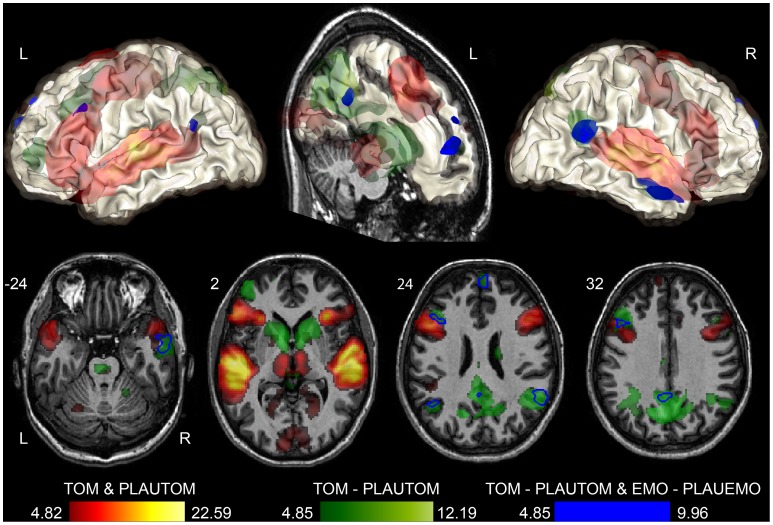
Overlap between emotional and ToM sentence processing (in blue). Three-plane views and surface renderings of the significant activations during language comprehension (conjunction of PLAUTOM and TOM, in warm colors) and ToM sentence comprehension) as compared with a plausibility judgment task on sentences of comparable complexity (TOM - PLAUTOM, in green). The overlap between the TOM task and the emotional sentence classification task (EMO), relative to their matched plausibility judgment tasks (PLAUTOM and PLAUEMO) is rendered or contoured in blue (conjunction analysis, *p*<0.05 FWE). The functional data (SPM t-map) are overlaid on the mean grey matter image of the stereotaxic template (T-80TVS, MNI space). The functional activation threshold was set at *p*<0.05, FWE correction for multiple comparisons. The conjunction was masked so as to include only areas that also differ between the EMO and GRAM tasks (at *p*<0.0001, uncorrected).

The conjunction of TOM and PLAUTOM activations (Figure 1, in red) revealed significant bilateral activations in the superior temporal gyrus, from the pole to the posterior verticalization of the Superior Temporal Sulcus (STS), in the inferior frontal gyrus, extending into the adjacent precentral gyrus or anterior insula, and in the calcarine fissure (voxel-level threshold: *p*<0.05, FWE correction for multiple comparisons). The left sensorimotor cortex, thalami, anterior globi pallidi and the SMA/preSMA region were also activated by both tasks. A cluster of left dmPFC was also significantly activated (x = −10, y = 60, z = 34, t = 6.68 with 52 voxels, visible on the slice at z = 32 in Figure 1).

#### Areas involved in ToM

The contrast between TOM and PLAUTOM (Table 3, Figure 1) revealed significant bilateral activations of the TPJ (voxel-level threshold: *p*<0.05, FWE correction). This location corresponded to the portion of the angular gyrus that is situated at the axial height level of the point of verticalization of the Sylvian fissure, which constitutes the anterior landmark for the separation of the temporal and parietal lobes. The activation of the TPJ was more significant and more extended in the right hemisphere. A strong activation cluster spanned the precuneus and pCC. The dorsal mPFC was also activated in two separate parts, in the left and right hemisphere, with the right hemisphere cluster being slightly lower and larger. The anterior middle temporal gyrus (MTG) of the right hemisphere, below the STS, was also recruited by TOM compared with PLAUTOM. A cluster of 3 voxels at MNI coordinates x = 2, y = 56, z = −8 (t = 5.14) and a single voxel at x = 2, y = 54, z = −12 (t = 4.86) were found in the anterior vmPFC region (a-vmPFC).

**Table 3:** Stereotaxic peak coordinates (MNI space, coordinates in mm, p<0.05 FWE, clusters with more than 10 voxels) for the theory of mind task contrast and conjunction between EMO and TOM tasks, relative to their reference (PLAUEMO and PLAUTOM).

Anatomical region	x		y		z	*N* voxels	*t*
*[TOM – PLAUTOM]*							
Bilateral precuneus	−6		68		38	6616	12.19
	8		−60		30		9.88
Bilateral intraparietal sulcus	−36		−52		42		10.80
	18		−64		54		5.91
Bilateral TPJ	−44		−60		24		6.15
	52		−54		24	1163	9.63
Bilateral caudate head	−8		8		2	4165	10.07
	8		6		0		8.86
Left anterior middlefrontal gyrus	−44		58		2	387	8.61
Bilateral posterior inferiorfrontal sulcus	44		22		32	24	5.13
	−44		28		32	1873	7.96
Bilateral posterior superior frontal sulcus	−32		6		58		7.48
	32		6		56	780	6.42
Right middle temporal gyrus	62		−4		−24	588	7.42
Bilateral dmPFC	4		64		22	166	6.33
	−4		52		38	23	5.32
SMA	4		2		56	199	6.06
Right postcentral sulcus	40		−26		36	194	5.73
Cerebellum	22		−46		−26	28	5.68
Left central sulcus	−42		−18		56	11	5.10
*[EMO – PLAUEMO] & [TOM – PLAUTOM]*							
Bilateral posterior cingulate cortex	−6	−52		30		102	9.96
Bilateral TPJ	54	−50		24		322	9.13
	−44	−58		22		65	5.66
Right anterior middle temporal gyrus	62		−6		−22	359	7.40
Left posterior inferior frontal sulcus	−46		26		28	128	6.86
Bilateral dmPFC	4		62		22	164	6.17
	−4		52		38	22	5.32

The *t* statistic for each peak, and the size of the corresponding activation cluster in number of voxels (8 mm^3^ volume) are also presented.

In the left middle frontal gyrus, two separate activations were found, one near the anterior convexity of the frontal lobe, and the second one just before the junction of the inferior frontal sulcus with the precentral sulcus. Strong activation foci were found in the caudate nuclei, in a bilateral activation cluster that extended posteriorly into the thalami, and into the anterior putamen. Activations were also present in the left intraparietal sulcus and in the depths of the right postcentral sulcus, the bilateral posterior end of the superior frontal sulcus, and in sensory or motor regions such as the central sulcus and SMA.

#### Areas active during both ToM and emotional sentence processing

Among the areas activated during the TOM – PLAUTOM contrast described above, the conjunction analysis between [TOM – PLAUTOM] and [EMO – PLAUEMO] revealed significant overlap at the level of the right MTG, bilateral TPJ, pCC, and both clusters of dmPFC (voxel-level threshold: *p*<0.05, FWE correction). Overlap was also seen at the level of the inferior frontal sulcus. These areas are highlighted by the blue contours in Figure 1, and listed in Table 3. The a-vmPFC voxel at x = 2, y = 54, z = −12 was included in the conjunction analysis.

#### Differences between ToM and emotional sentence processing

**Figure 2:**
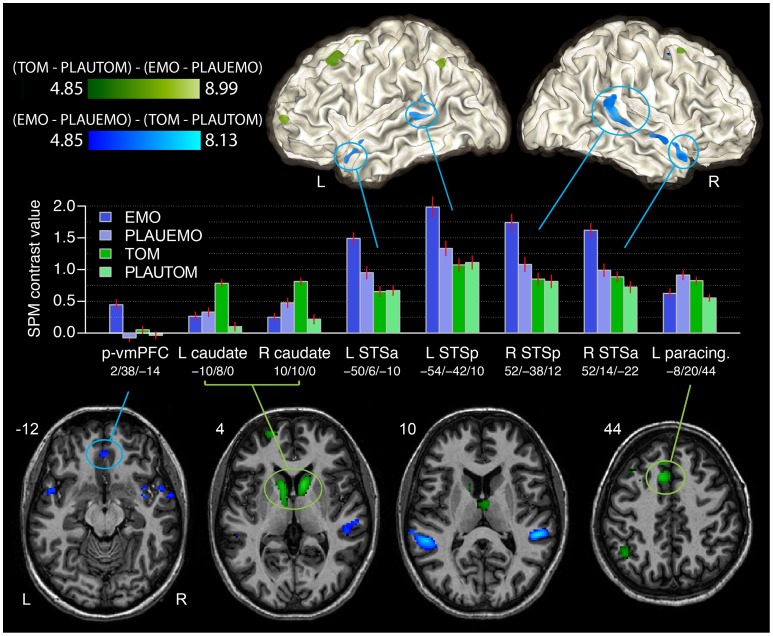
Differences between emotional and ToM sentence processing, as assessed by comparisons between EMO (blue shades) and TOM (green shades), relative to their corresponding plausibility judgment tasks (PLAUEMO and PLAUTOM). The functional data (SPM t-map) are overlaid on a representative subject in the MNI space, on white matter surface and on axial slices in neurological orientation. The accompanying plots (mean ± SEM) present the SPM contrast values (relative to beep-detection baseline) during the two pairs of sentence comprehension tasks in the regions more active during EMO than TOM (blue lines), and during TOM compared with EMO (green lines), contrasted to the PLAUEMO and PLAUTOM reference tasks. The voxel-wise functional activation threshold was set at *p*<0.05, corrected for multiple comparisons. The [EMO – PLAUEMO] – [TOM – PLAUTOM] contrast, showing regions more active during emotional than ToM speech processing, was masked inclusively by the EMO – GRAM contrast (at *p*<0.0001, uncorrected). The reverse contrast ([TOM – PLAUTOM] – [EMO – PLAUEMO]) was masked inclusively by the TOM – PLAUTOM contrast (at *p*<0.0001, uncorrected).

Relative to their plausibility judgment tasks, ToM sentence classification elicited greater activity than emotional sentence classification in the caudate nuclei and adjacent putamen and thalamus, and the paracingulate cortex (voxel-level threshold: *p*<0.05, FWE correction). Other areas included the bilateral posterior superior frontal sulcus and neighboring middle frontal gyrus, the left anterior superior frontal sulcus near the convexity of the fontal lobe, as well as the left intraparietal sulcus (Figure 2, Table 4).

**Table 4:** Stereotaxic peak coordinates (MNI space, coordinates in mm) for the differences between EMO and TOM tasks, relative to their references (PLAUEMO and PLAUTOM).

Anatomical region	x	y	z	*N* voxels (8 mm^3^)	*T*
*[TOM – PLAUTOM] – [EMO – PLAUEMO]*					
Bilateral caudate nucleus	10	10	0	405	8.99
	−10	8	0	422	7.81
Left paracingulate cortex	−8	20	44	236	7.28
Left intraoccipital sulcus	−34	−78	38	21	6.66
Left anterior superior frontal sulcus	−20	62	4	57	6.22
Left intraparietal sulcus	−44	−54	44	101	6.20
Left superior frontal sulcus	−28	6	60	81	6.17
Bilateral middle frontal gyrus/superior frontal sulcus	−32	22	52	154	5.72
	34	12	56	37	5.52
*[EMO – PLAUEMO] – [TOM – PLAUTOM]*					
Bilateral posterior superior temporal sulcus	52	−38	12	340	8.13
	−54	−42	10	217	7.52
Bilateral anterior *planum polare/insula*	38	4	−16	66	7.47
	−36	2	−18	11	5.48
Left posterior *planum temporale*	−58	−42	24	48	6.53
Bilateral anterior STS	52	14	−22	75	6.33
	60	−8	−6	57	6.04
	−50	6	−10	70	6.02
vmPFC	2	38	−14	25	5.57

The T statistic for each peak, and the size of the corresponding activation cluster in number of voxels (8 mm^3^ volume) are also presented.

Conversely, the EMO task was associated with greater activity than TOM in the anterior and posterior superior temporal sulcus bilaterally, the anterior and medial planum polare or adjacent insula, and in a more posterior vmPFC region (p-vmPFC, Figure 2, Table 4).

### Regional Analyses

**Figure 3:**
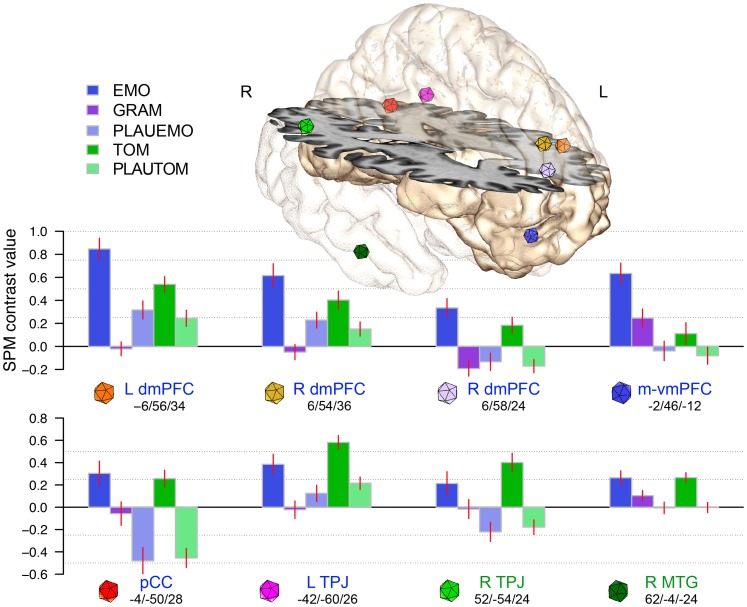
Response profile of Medial-network nodes (mean ± SEM). The BOLD contrast values during the EMO, GRAM, and PLAUEMO tasks (blue shades), and TOM and PLAUTOM tasks (green shades), relative to beep-detection baseline, were extracted in the 6 regions of the Medial network defined in a previous study using the EMO and GRAM tasks (dmPFC, vmPFC, pCC and Left TPJ; blue labels, see [5]). The coloured spheres (4 mm radius) indicate the regions-of-interest. We added the two mentalizing regions of the right hemisphere (R TPJ and R MTG; green labels), uncovered by the addition of the TOM, PLAUTOM and PLAUEMO tasks.

The BOLD signal variations across the 5 tasks in the 6 ROIs that constituted the previously described Medial network [5] are shown in Figure 3. The statistical analyses (Table 5) revealed that, except in the vmPFC (blue dot), all the regions of the Medial network were active during TOM compared with beep-detection baseline, or during TOM compared with PLAUTOM.

**Table 5:** One-sample t-tests assessing activations during TOM, PLAUTOM or PLAUEMO, and paired two-sample t-tests assessing the TOM – PLAUTOM differences in the 6 Medial network regions.

Region	Degrees of freedom	TOM			PLAUTOM			PLAUEMO			TOM – PLAUTOM		
		t	p-value	Adj. p-value (Holm)	t	p-value	Adj. p-value (Holm)	t	p-value	Adj. p-value (Holm)	t	p-value	Adj. p-value (Holm)
L dmPFC -6 56 34	41	8.06	5×10^−10^*	3×10^−9^*	3.51	0.001*	0.004*	4.04	2×10^−4^*	0.001*	5.64	1×10^−6^*	6×10^−6^*
R dmPFC 6 54 36	41	5.18	6×10^−6^*	3×10^−5^*	2.45	0.019*	0.038*	3.34	0.002*	0.007*	3.80	5×10^−4^*	9×10^−4^*
R dmPFC 6 58 24	41	2.61	0.013*	0.025*	−2.97	0.005*	0.015*	−1.79	0.081	0.243	5.08	9×10^−6^*	3×10^−05^*
m-vmPFC -2 46 -12	41	1.17	0.251	0.251	−1.11	0.275	0.275	−0.47	0.643	0.643	1.98	0.054	0.054
pCC -4 -50 28	41	3.41	0.001*	0.004*	−5.28	5×10^−6^*	3×10^−5^*	−4.01	2×10^−4^*	0.001*	8.25	3×10^−10^*	2×10^−9^*
L TPJ -42 -60 26	41	9.41	8×10^−12^ *	5×10^−11^*	3.88	4×10^−4^*	0.002*	1.72	0.094	0.243	8.19	4×10^−10^*	2×10^−9^*

Within each task, a Holm-Bonferroni procedure was applied to correct for multiple comparisons (6 null-hypotheses). Asterisks indicate raw or adjusted p-value below 0.05. The m-vmPFC region did not show any significant result, although a trend is present in the TOM – PLAUTOM comparison.

During the plausibility judgment tasks, the left TPJ showed a significant activation during the PLAUTOM, but not the PLAUEMO judgment task (Table 5), likely related to a difference in syntactic complexity between these two tasks. The uppermost left and right dmPFC regions (orange dots) were recruited during both PLAUEMO and PLAUTOM. Conversely, the inferior right dmPFC tended to display deactivations during these two tasks, reaching statistical significance only during PLAUTOM. The pCC displayed significant deactivations during both plausibility tasks. No significant change was detected in the vmPFC during PLAU tasks.

**Figure 4:**
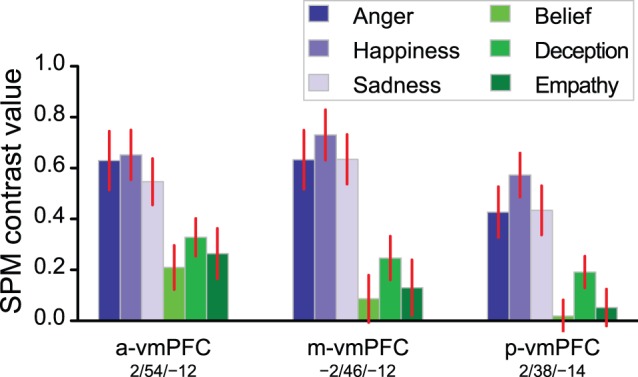
Average BOLD response of 3 vmPFC regions-of-interest to the 3 different conditions of EMO or TOM (mean ± SEM). Left: significant region from the EMO and TOM conjunction (a-vmPFC); Centre: region of the Medial network (m-vmPFC); Right: significant region from the EMO – TOM comparison (p-vmPFC).

Because a greater involvement of the vmPFC in affective compared to cognitive ToM is reported in the literature [10], we compared the 3 conditions of TOM (*Belief, Deception, Empathy*) in 3 ROIs located within this region. The most anterior ROI was the a-vmPFC peak of the conjunction analysis, followed by the Medial network ROI (m-vmPFC), and p-vmPFC peak of EMO minus TOM comparison (Figure 4). *Empathy* did not differ significantly from *Belief* (a-vmPFC: *p* = 0.46, m-vmPFC: *p* = 0.64, p-vmPFC: *p* = 0.58) or *Deception* (*p* = 0.38, *p* = 0.24, *p* = 0.09). *Deception* differed from *Belief* in the m-vmPFC and p-vmPFC (*p* = 0.01 and *p* = 7×10^−4^ respectively, a-vmPFC: *p* = 0.07).

## Discussion

This study demonstrated that the emotional component of sentence comprehension and ToM recruit both common and specific areas. The ROI based analyses confirmed that the coherent network of 6 medial and angular regions that is recruited during the emotional component of sentence comprehension (the Medial network defined in [5]) is also active during a task probing the neural bases of ToM. The vmPFC ROI of this network (m-vmPFC) was the only exception, and this may be due to the particular status of the vmPFC with respect to cognitive and affective processes. The exploratory whole-brain analyses accordingly detected a spatial overlap between emotional and ToM sentence processing in regions that are important for mentalizing, such as the TPJ bilaterally, the posterior cingulate cortex, the right MTG and the left and right dorsal mPFC. In the vmPFC area, a trend towards activation during TOM was found in a more anterior part (a-vmPFC). A significantly higher activity during emotional compared with ToM sentence processing was nonetheless observed in the posterior part of the vmPFC (p-vmPFC, Figure 3).

Prior to evaluating the role of this region in mentalizing in the light of the differences between the EMO and TOM tasks, and then interpreting the involvement of the caudate and other brain regions during TOM compared with EMO, the discussion will first deal with the overlap between ToM and emotional sentence processing networks, and the relationships between the ToM network and sentence comprehension.

### Overlap between ToM and the Emotional Component of Sentence Comprehension

In accordance with our starting hypothesis, the results confirmed that a same coherent network contributes to both ToM and emotional components of sentence comprehension. The distributed network shared by ToM and emotional components of sentence processing displays similarities with the default mode network, especially the dmPFC subsystem and core components [11]. This network has been associated with several more or less overlapping cognitive functions, such as story comprehension [12], self-projection during mind wandering, ToM, prospection and episodic memory [13], [14], or semantic processing [15]–[17]. Within this network, the TPJ region has also been associated with bottom-up attention orienting [18], [19]. The activity of this network, although it is clearly involved in mental representation processing, may not be restricted to mentalizing.

Nonetheless, studies comparing affective and cognitive ToM cartoons [7], [8] have reported a neural overlap in the same regions as those evidenced in the present study between EMO and TOM (Figure 1). Importantly, solving the EMO task did not explicitly require ToM processes, as emotion recognition from affective prosody or words is sufficient and the instructions focused on the sentences. In contrast, the TOM task explicitly required mentalizing. The results thus suggest that an additional process akin to ToM occurs during the EMO task, as part of the emotional component of affective sentence comprehension. In the EMO task debriefing interviews, a large majority of the participants answered positively to questions about their reliance upon the simulation of the speakers’ perspective (simulation-theory) and/or their social knowledge (theory-theory) [5].

Right hemisphere TPJ and MTG regions, which had not been sampled in the previous study, were evidenced in the present study as part of the regions shared by both EMO and TOM tasks. This difference is explained by the response profiles of the right TPJ and MTG peaks, which were either deactivated or not activated during PLAUEMO. Compared with the GRAM reference task, which elicited a slightly greater activity in these regions, the use of PLAUTOM increased the sensitivity of the subtraction analysis (Figure 3). The fact that both these regions were found in TOM – PLAUTOM as well as in EMO – PLAUEMO (Figure 1) is fully consistent with their reliable involvement in ToM tasks [20], as well as with their functional connectivity pattern [21].

Although the “Medial network” did not incorporate any lateral prefrontal ROIs, the left posterior inferior frontal sulcus was selected by the conjunction analysis. This region, however, has been associated with sentence processing [22] as well as cognitive control [23], [24]. Accordingly, a possible reason why this region supports both emotional and ToM sentence comprehension is that such sentences would require a more intensive processing than reference sentences.

### Language and ToM

The results support the view that verbal “fictional third-person stories” are appropriate stimuli for the functional imaging of ToM [25]. In the present experiments, we used spoken sentences, which constitute shorter stimuli than false-belief stories and fit within an event-related design. The protocol was sensitive enough to enable the detection of ToM-related activations, and separate them from language-related activations (Figure 1). The debriefing also suggested that the participants took into account linguistic cues when classifying the ToM sentences, particularly mental state words. This further confirms that one can rely on this set of linguistic tools for mental state representation when studying ToM with functional imaging [25], [26].

Some of the ROIs of the Medial, ToM-related network, namely the dmPFC and left TPJ appeared to respond as well to sentence processing during plausibility judgments, albeit to a lesser extent (Figure 3). Both regions may thus participate of the interface between the ToM and the language-related networks, which seems necessary for accurate verbal communication, for instance in the case of irony or indirect requests [27]–[29]. The dmPFC is more strongly connected with the IFG during the processing of ironic as opposed to literal texts, a contrast that also evidences the involvement of ToM regions in the pragmatic aspects of sentence comprehension [29]. The engagement of the dmPFC region during sentence comprehension is further suggested by the fact that, in the whole brain analyses, a significant cluster was found just next to this left dmPFC ROI in the conjunction between TOM and PLAUTOM. This result is consistent with previous observations of activity in the dmPFC region during language tasks involving series of sentences, in the absence of ToM and in relation with text-coherence building or reasoning [30], [31]. During plausibility judgments, the left TPJ was significantly active only with the more complex PLAUTOM sentences. Conversely, the right TPJ showed deactivations during spoken sentence comprehension, thereby appearing more specific of mental-state representation [20] than its left counterpart during the processing of either emotional or ToM sentences.

Finally, the bilateral activations of the anterior and posterior STS that were found in the comparison of EMO and TOM, relative to the plausibility judgment tasks (Figure 2), may be associated with the processing of affective prosody, which was present only during the EMO task (Figure 2). In our previous analyses of the functional connectivity during the EMO task [5], both these STS regions were included in the Perisylvian, speech-processing networks, as opposed to the Medial, ToM-related network. We hypothesize that the anterior STS regions are involved in the analysis of the speech signal, while the more posterior regions are involved in the integration of the extracted prosodic information with emotional, syntactic and semantic processes [4].

### Emotional Speech, ToM and the Ventromedial Prefrontal Cortex

The vmPFC displayed a complex behavior (Figure 4). The activity was larger during EMO in all 3 vmPFC ROIs. Nonetheless, in the whole brain analyses, with a conservative voxel-wise threshold, and relative to the two plausibility judgment tasks, EMO was significantly more active than TOM only in the p-vmPFC. At the same threshold, the TOM task elicited a significantly greater activity than PLAUTOM only in the a-vmPFC. One can conclude that, as a whole, the vmPFC may be significantly, but marginally involved during ToM sentence processing compared with plausibility judgments. This trend for an activation of the anterior vmPFC during TOM compared with PLAUTOM is in line with the fact that the vmPFC is functionally connected [5], [11] and frequently co-activated (http://neurosynth.org/seeds/-4_48_-12 [32]) with the network of ToM regions: changes in the activity of this network may thus be reflected in the vmPFC.

Conversely, the differential involvement of the p-vmPFC during the EMO and TOM tasks is consistent with the literature on affective and cognitive ToM [7], [9], [10]. This appears as a likely consequence of the focus on emotional material in the EMO task. Brothers and Ring had distinguished between the “hot and cold aspects of representation of mind”, with the phylogenetically older hot aspects originating from the fact that the intentions of the observed can have important social and emotional consequences for the observer [33]. The fact that the EMO sentences, but not the TOM sentences, were play-acted – with the presence of congruent affective prosody - made the EMO task hotter than the TOM task (including the TOM *Empathy* condition, which involved the mental states of absent and unfamiliar others, without a direct focus on emotions).

It has been proposed that the vmPFC would link decisions or situations with their emotional consequences, and may mark mental representations with affective information in the particular context of mentalizing [10], [34]. Accordingly, during the emotional sentence classification task, the vmPFC might incorporate online-generated information coming from emotional brain regions, especially the emotional prosody processing systems of the STS, into a broader emotional mental-state attribution process that would integrate all the information extracted from the sentence. In the particular context of isolated sentences, the presence of such affective information, rather than the object of the mentalizing (epistemic or emotional mental states), might be the strongest determinant of the involvement of the vmPFC during ToM: this would explain why the TOM *Empathy* condition was not especially associated with increased activity in the vmPFC ROIs (Figure 4).

Under this hypothesis, the amygdala, given its importance in emotional processing and its connections with the vmPFC [35], might also be expected to interact with the vmPFC during the EMO task. Although we have previously reported an increased activation during EMO compared with GRAM in the amygdala [5], we did not detect a significant difference between EMO and TOM, relative to PLAU and PLAUTOM in this region.

### ToM Sentence Processing and Executive Function

The TOM task sentences involved more complex mental states than the EMO task. Strikingly, the region that showed the greatest difference in terms of hemodynamic activity during TOM, compared with EMO, was the caudate nucleus, bilaterally. Activations of the caudate nucleus are sometimes reported in a sentence-processing context, for instance during metaphor comprehension [36] or when reading sentences in a non-native language [37]. Besides, deficits in both affective and cognitive ToM have been described in Parkinson’s disease, a condition in which the striatum is affected [38]–[41]. The review by Poletti et al. concludes that cognitive ToM is the mainly concerned component, while the affective component could be impaired later on during the course of the disease [42]. The cognitive alterations associated with Parkinson’s disease have been described as “predominantly executive”, affecting the mechanisms that allow several simultaneous processes to coexist efficiently during complex cognitive tasks [43]. Accordingly, the early effect of the disease on ToM could be mediated in part by a negative effect of dopamine depletion in the dorsolateral frontostriatal circuit on executive functions performed by the prefrontal cortex [42], such as working memory or inhibition, which are important for solving false-belief ToM tasks [44].

If impairments in ToM processing can occur as a consequence of executive dysfunction caused by impairment of fronto-striatal circuits, then the widespread activation of the striatum observed during TOM, compared with EMO, could reflect the executive processes supporting the TOM task. Several regions known to be involved in executive function were also activated along with the caudate nuclei during the TOM task: we found a bilateral increase in activity during TOM compared with EMO in the posterior superior frontal sulcus, paracingulate cortex and intraparietal sulcus (Figure 2, Table 4). The paracingulate region is associated with response selection and conflict monitoring [45], and shows connectivity with the caudate in anatomical and functional terms [46]–[48]. The results suggest that the different sentence classification tasks imposed different constraints on the executive processes supporting ToM or sentence comprehension processes, thus modulating the activity in executive neural networks. TOM, of all the sentence classification tasks involved in the present study, was the hardest to perform, with a greater error rate and longer response times than PLAUTOM.

### Study Limitations

When interpreting these results, it is also important to keep in mind the limitations of the experimental design. We had to acquire the data for the EMO and PLAUEMO tasks on two different sessions, whereas the TOM and PLAUTOM tasks were acquired on the same day. It is therefore not possible to rule out that systematic intersession differences could have affected the sensitivity or the outcome of the relative comparison of the EMO and TOM tasks.

The second point concerns differences in performances across tasks. Although the PLAU tasks eliminated differences in terms of stimuli length and grammatical construction, the EMO and TOM sentences were different on average in terms of the response times and accuracy as a direct consequence of the more complex situations that had to be used in the TOM task. Note that adjusting for response times in second-level analyses did not affect the pattern of significant results.

### Conclusion

This neuroimaging study used sentence classification tasks based either on emotions or type of mental contents to compare the neural correlates of emotional and mental-state-related components of speech comprehension. A network of shared functional areas was found, with classical ToM regions being recruited in both ToM and emotion classification tasks. This suggests an intricate relation between emotion recognition and the inference of the cognitive states of others, with ToM processes being automatically involved during emotional sentence comprehension. This automaticity is suggested by the fact that the participants were instructed to classify sentence contents, not the emotional states of the speaker. Compared with the ToM task, emotional sentence classification was associated with increased activity in the bilateral posterior and anterior STS, likely in relation with the processing of emotional prosody cues, as well as in the p-vmPFC. Previous research on affective and cognitive ToM [10] indicates that this latter region would be involved in the representation of emotional mental states. Accordingly, in the present study, the vmPFC was more active in an affective sentence classification task, in the presence of emotional material (words and prosody), compared with a colder task in which one had to represent the minds of absent, unfamiliar characters of sentences which were read in a neutral way.

## Supporting Information

Materials S1 **List of the sentences used in the 5 tasks (TOM, PLAUTOM, EMO, PLAUEMO, GRAM).** Each task comprised 48 sentences, separated in 2 fMRI runs of 24 sentences. The numbers of the PLAUTOM and PLAUEMO sentences match those of the sentences of the TOM and EMO sentences they were derived from. The bold letters in PLAUTOM and PLAUEMO highlight the incongruent words in the implausible sentences. For the GRAM task, the numbers at the end of the lines indicate the grammatical person (1^st^, 2^nd^ or 3^rd^).
(PDF)
