# Supplementary material for: A Shared Neural Substrate for Mentalizing and the Affective Component of Sentence Comprehension
Source: PLoS One. 2013 Jan 16;8(1):e54400. doi: 10.1371/journal.pone.0054400 (PMC3547007; doi:10.1371/journal.pone.0054400)
Supplement: Materials S1 — List of the sentences used in the 5 tasks (TOM, PLAUTOM, EMO, PLAUEMO, GRAM). Each task comprised 48 sentences, separated in 2 fMRI runs of 24 sentences. The numbers of the PLAUTOM and PLAUEMO sentences match those of the sentences of the TOM and EMO sentences they were derived from. The bold letters in PLAUTOM and PLAUEMO highlight the incongruent words in the implausible sentences. For the GRAM task, the numbers at the end of the lines indicate the grammatical person (1st, 2nd or 3rd). (PDF) [file pone.0054400.s001.pdf]

TOM

Belief:

1. Sa copine ne lui parle pas de leurs prochaines vacances car elle pense qu'il va la quitter

His girlfriend does not talk to him about their next holidays because she thinks that he is going to leave her.

2. L'adolescent attend patiemment sa copine à la sortie du lycée car il ignore que son cours a été annulé

The adolescent patiently waits for his girlfriend at the entrance of the high school because he is not aware that the lecture has been cancelled.

3. Il s'imagine la convaincre mais il a oublié qu'elle connaît toute l'histoire.

He imagines convincing her but he forgot she knows the whole story.

4. Son ami pense qu'elle aime les intellectuels parce qu'elle a toujours un bouquin avec elle.

Her friend thinks that she likes intellectuals because she always has a book with her.

5. Elle est sûre que son ex-mari n'aura pas le courage de répondre à sa lettre.

She is sure that her ex-husband will not have the courage to answer her letter.

6. L'enfant est sûr que le magicien a fait disparaître l'oiseau.

The child is sure that the magician made the bird disappear.

7. Comme c'est une lectrice de Libération, il pense savoir pour qui elle a voté.

Since she is a reader of Liberation newspaper, he thinks he knows who she voted for.

8. Malgré leur comédie, le détective ne croit pas une seconde qu'ils soient amants

Despite their play-acting, the detective does not believe for a second that they are lovers.

Deception:

9. Sa réunion annulée, elle dit pourtant à son mari qu'elle va travailler tard

Her meeting cancelled, she however tells her husband that she is going to work late.

10. Malgré l'odeur, il assure à sa cliente que son poisson est frais.

Despite the smell, he assures his client that his fish is fresh.

11. Les valises pleines de fausses montres, le touriste assure au douanier qu'il n'a rien à déclarer.

The luggage being full of fake watches, the tourist assures the customs officer that he has nothing to declare.

12. Anticlérical, il loue le pape dans son discours électoral pour attirer les catholiques.

Anticlerical, he praises the pope with his electoral speech to attract the Catholics.

13. Surpris par son prof pendant l'examen, il lui affirme qu'il ne regardait pas la copie de son voisin.

Surprised by his teacher during the examination, he maintains that he was not looking at his neighbour's paper.

14. La femme se maquille pour cacher son bronzage avant de rencontrer le médecin de la sécurité sociale.

The woman puts on make-up to hide her suntan before meeting the social security doctor.

15. Il s'est mis à rire avec les autres pour cacher qu'il n'avait pas compris la blague.

He started laughing with the others to hide that he did not catch the joke.

16. Bien qu'elle ait l'air ridicule, la vendeuse lui affirme que la robe lui va très bien.

Though she looks ridiculous, the seller maintains that the dress suits her very well.

Empathy:

17. Son bébé a l'air si calme sur la photo qu'elle se sent sereine.
Her baby looks so calm on the photo that she feels serene.
18. Après leur conversation sur les travaux de la maison, son compagnon aimerait être avec elle pour l'aider.
After their conversation about the works on the house, her companion would like to be with her to help her.
19. Il est révolté par le récit de sa sœur qui a été renvoyée sans motif de son emploi.
He is appalled by the story of his sister who has been fired from her job without reason.
20. L'oncle a offert aux enfants la même glace à la fraise pour qu'ils ne soient pas jaloux.
The uncle offered the two children the same strawberry ice cream so they would not be jealous.
21. En voyant son visage souriant à son arrivée, elle sent qu'il partage le plaisir de cette rencontre
On seeing his smiling face when arriving, she feels that he shares the pleasure of this meeting.
22. Quand ils annoncent au patient que sa tumeur est bénigne, les médecins sont contents de voir le soulagement du malade
When they announce to the patient that his tumour is benign, the doctors are pleased to see the patient's relief.
23. Les policiers ne verbalisent pas le conducteur qui a grillé un feu en emmenant sa femme enceinte à la clinique
The policemen do not fine the driver who ran the red light driving his pregnant wife to the clinic.
24. Elle est heureuse de voir la joie des pompiers qui ont sauvé l'homme de l'incendie.
She is happy to see the joy of the firemen who rescued the man from the fire.

Belief:

25. Parce que le garagiste est une femme, on la prend toujours pour la secrétaire
Because the car mechanic is a woman, everyone thinks she is the secretary.
26. Sa plaisanterie sournoise lui laisse penser que son voisin se doute qu'elle a une liaison.
His insidious joke makes her think that her neighbour suspects that she has an affair.
27. A cause du déguisement qu'elle porte, le patron du café lui a indiqué les toilettes des hommes
Because of her disguise, the cafe's landlord directed her to the men's toilets
28. Après ce qui s'est passé entre eux, elle n'envisage pas qu'il aura l'audace de la revoir
After what happened between them, she does not think that he will have the audacity to meet her again.
29. Aujourd'hui, premier jour des soldes, elle sait où trouver sa sœur qui est très coquette.
Today, first day of the sales, she knows where to find her sister who is very stylish.
30. Avec sa patte de lapin dans la poche, il est sûr de gagner la course.
With his rabbit-foot in his pocket, he is sure to win the race.

31. Devant son air solennel au moment de lui parler, elle s'attend cette fois à ce que son mari lui dise la vérité.
Considering his solemn air as he is about to speak to her, she expects that this time, her husband will tell her the truth.
32. Malgré les explications qu'il lui a données, sa fille est sûre qu'il va la punir.
Despite the explanations he gave her, her daughter is sure that he is going to punish her.

Deception:
33. La directrice adjointe félicite chaleureusement son collègue qui a obtenu le poste de directeur après avoir tout fait pour l'en empêcher.
The assistant director warmly congratulates her colleague who was appointed as director, though she had attempted everything to stop him.
34. Pour jouer à la roulette le mineur montre une fausse carte d'identité à l'entrée du casino.
To play roulette, the minor shows a fake identity card at the entrance of the casino.
35. L'antiquaire présente une copie de tableau à l'amateur d'art comme s'il s'agissait d'un original.
The antique dealer shows a copy of a painting to the art amateur as if it was an original.
36. Pour conserver leur admiration, il n'a pas dit à ses amis qu'il avait perdu son procès.
To keep their admiration, he did not tell his friends that he lost his trial.
37. Pour séduire la jeune femme, l'homme garde son chapeau qui cache sa calvitie.
To charm the young woman, the man keeps wearing his hat to hide his baldness.
38. Pour ne pas goûter le gâteau de sa mère, il lui dit qu'il est malade.
To avoid tasting his mother's cake, he tells her he is sick.
39. Arrêtée pour avoir grillé un feu, la conductrice affirme au policier qu'elle est passée au vert.
Arrested for running a light, the driver tells the policeman that she went when the light was green.
40. Alors qu'il est salarié, il affirme qu'il est au chômage pour avoir une réduction.
Although he is employed, he says he is out of work to get a discount.

Empathy:
41. Pour la mettre à l'aise, il fait comme s'il ignorait qu'elle n'était pas invitée
To put her at ease, he acts like he did not know that she was not invited.
42. Pour ne pas gâcher la soirée de Pierre, personne ne lui a dit qu'il avait chanté comme une casserole.
To not ruin Pierre's party, nobody told him that he sang flat.
43. Elle appréhende sa déception lorsque son fils connaitra les résultats de l'élection.
She dreads his disappointment when her son will hear about the election results.
44. Quand son conjoint a eu la nouvelle, elle l'a senti tellement joyeux qu'elle lui a sauté au cou !
When her companion got the news, she felt he was so happy that she flew into his arms!
45. Comme la cliente avait les bras chargés de paquets, il lui a tenu la porte
Since the client had his arms full with parcels, he held the door.
46. La vétérinaire parle doucement au chaton apeuré.
The veterinarian speaks softly to the frightened kitten.

47. Elle propose à son invitée de se reposer dans sa chambre car elle la trouve fatiguée
She proposes her guest to rest in her bedroom because she finds her tired.
48. Pour ne pas l'impressionner, l'homme s'accroupit quand il parle au petit garçon
To not impress him, the man squats when he talks to the little boy.

PLAUTOM

1. L'oiseau ne s'approche pas de la branche car il voit que les **carottes** la secouent
*The bird does not come close to the branch because he sees that the **carrots** are shaking it*.
2. La lune cache complètement le soleil d'été car il s'avère que l'éclipse a commencé.
The moon completely hides the summer sun because it turns out that the eclipse has begun.
3. Les bourgeons commencent à s'ouvrir mais on voit que les pucerons les attaquent.
The buds begin to open but we see that the aphids attack them.
4. On voit que les vignes ont le mildiou parce que les feuilles ont des tâches noires en surface.
We see that the vines have mildew because the leaves have black stains on the surface.
5. C'est sur que la voiture n'aura pas la puissance pour monter la côte
It is sure the car will not have enough power to climb the hill.
6. Il est évident que la chaleur a fait craqueler la terre
It is obvious that the heat caused the ground to crack.
7. Comme c'est une toile de maître, il reste à savoir à qui elle a été **chantée**
*Since it is a master's painting, the question is to know to who it was **sang** to*.
8. Malgré leurs belles couleurs, il ne semble pas que ces champignons soient comestibles.
In spite of their nice colours, it does not look like these mushrooms are edible.
9. Le marathon achevé, la douleur indique au coureur que ses muscles vont être **rédigés** ce soir.
*The marathon over, the pain tells the runner that his muscles will be **redacted** tonight.*
10. Malgré la crise les transactions montrent aux économistes que le **chiot** est parti
*Despite the crisis, the transactions show the economists that the **puppy** is gone.*
11. Face aux étals plein de fruits mûrs, il apparaît aux touristes que le soleil n'a pas du manquer.
Facing the stall full of ripe fruits, it occurs to the tourists that sun was not missed.
12. Énergique, il lance la paille dans la grange pour nourrir les vaches.
Energetic, he throws the hay in the grange to feed the cows.
13. Poussés par le vent pendant la soirée, les rares nuages indiquent que la grêle ne menacera pas les cultures.
Pushed by the wind during the evening, the rare clouds indicate that the hail will not threaten the farming.
14. Une analyse se justifie pour identifier la bactérie avant de traiter l'eau de l'hôpital
An analysis is justified in order to identify the bacteria before treating the hospital water.
15. La pluie s'est mise à tomber avec force sans empêcher que l'incendie ne gagne la forêt

The rain began to fall heavily without preventing the fire from spreading to the forest.
16. Bien que le ciel soit très nuageux, la météo annonce que le soleil tapera très fort.
Despite the very cloudy sky, the weather forecast announces that the sun will beat down very hard.
17. Les feuilles ont l'air si sèches sur la plante qu'elle semble morte.
The leaves look so dry on the plant that it seems dead.
18. Après son exposition au musée d'Orsay la sculpture pourrait revenir à la collection du Louvre pour la compléter.
After the exhibition at the Orsay museum the sculpture could come back to the collection of the Louvre to complete it.
19. L'étal est garni de fruits d'importation qui ont été changés de place sans **casserole**
The stall is full of imported fruits that have been moved without ***saucepan****.*
20. La montagne offre au regard de magnifiques cimes enneigées pourvu qu'elles ne soient pas embrumées
The mountains offer a view over beautiful snow peaks as long as they are not misty.
21. En finançant de nouveaux spectacles en banlieue, on voit que la municipalité encourage l'accessibilité à la culture.
As it funds new spectacles in the suburbs, one can see that the local council supports the accessibility to culture.
22. Quand ils indiquent aux automobilistes que leur vitesse est excessive, les contrôles sont utiles pour limiter les mises en plis
When they tell the drivers that their speed is excessive, speed checks are useful in order to limit the folding-up.
23. Le journal n'évoque pas la tempête qui a dévasté le village en emportant de nombreux toits au loin
The newspaper does not mention the storm that devastated the village, blowing away many roofs.
24. Il est intéressant de regarder les transformations qui ont déclenché l'éclosion des **chaises**
It is interesting to watch the transformations that triggered the hatching of the ***chairs***.

25. Parce que les bonbons sont des friandises, on en achète souvent pour les **hélicoptères**
Because the sweets are candies, we often buy some for the ***helicopters****.*
26. La couleur rouge de cette fraise laisse penser qu'il est bientôt temps qu'on la cueille.
The red colour of this strawberry indicates that it will soon be time to pick it.
27. A cause du mauvais temps qui se déchaine, les rafales de vent emportent les panneaux de signalisation lumineux
Due to the bad weather that rages, the gusts of wind blow away the road signs.
28. Après le tremblement de terre qui a eu lieu ce matin, on ne prévoit pas qu'il y ait d'autres secousses à redouter
After the earthquake that happened this morning, we do not predict that there are other earth tremors to fear.
29. Aujourd'hui, premier jour d'automne, le vent fait tournoyer les feuilles qui sont tombées
Today, the first day of fall, the wind blows the fallen leaves around.
30. Avec ses nouveaux skis, il est prêt à faire du **vélo**
With his new skis, he is ready for ***cycling****.*

31. Devant les risques d'embouteillage au moment de partir en vacances, Bison futé demande à ce que les girafes reportent leur départ.
Considering the risk of traffic jams at the time of the holiday departures, the traffic monitoring service recommends that the giraffes postpone their departure.
32. Malgré les soins qu'on lui a donnés, il est évident que l'arbre va perdre ses feuilles
Despite the care we gave to it, it is obvious that the tree will loose its leaves.
33. Les goélands rappellent énergiquement leurs petits qui ont pris des forces nouvelles après avoir quitté le nid pour apprendre à **cuisiner**
The seagulls energetically call back their offspring who built up their new strength after they left the nest to learn how to ***cook.***
34. Pour participer au carnaval, il achète un gros nez de clown à l'entrée de l'avenue.
To participate in the carnival, he buys a big clown nose at the entrance of the avenue.
35. Le toboggan présente une faible pente à la fin de la descente comme s'il s'agissait d'une **piste de danse**.
The toboggan has a gentle slope at the end of the descent as if it was a ***dance floor***.
36. Pour protéger les ouvrages, il n'est pas conseillé aux libraires d'exposer les livres anciens
To protect the books, it is not advised to the booksellers to display the old books.
37. Pour accompagner cette viande rouge, on conseille un vin qui a du corps
To accompany this red meat, we advise a full-bodied wine.
38. Pour ne pas rayer l'objectif de la caméra, il faut qu'il soit protégé
To not scratch the lens of the camera, it needs to be protected.
39. Reconnues pour avoir déclenché une mobilisation, les élections montrent aux média que la **gastronomie** a progressé au Kenya
Recognized for having triggered a mobilisation, the elections show the media that ***gastronomy*** *has increased in Kenya.*
40. Alors que l'été est arrivé, on dirait que les arbres sont trop malades pour avoir des fruits
Although summer has arrived, it looks like the trees are too diseased to produce fruits.
41. Sans s'ouvrir complètement, la porte se débloque même s'il semblait qu'elle n'était pas utilisable
Without opening completely, the door unlocks even if it seemed that it was not usable.
42. Pour ne pas rater une crème de champignons, personne n'a dit qu'il fallait la mélanger comme une **poinçonneuse**
To not spoil a mushroom soup, nobody said that it had to be mixed like a ***punching-machine***.
43. Le brouillard envahit la plaine lorsque l'humidité imprègne l'air de la nuit.
The fog invades the plains when the humidity impregnates the night's air.
44. Quand la marée a atteint la digue, les vagues l'ont frappées si violemment qu'elles l'ont recouverte sur toute sa longueur.
When the tide reached the seawall, the waves hit it so violently that they covered it over its whole-length.
45. Comme le poirier avait des branches chargées de fruits, on lui a mis un tuteur.
Since the pear tree had branches overloaded with fruits, we put a support.
46. Ces immeubles résistent en souplesse aux séismes intenses.
These buildings resist flexibly to the intense earthquakes.

47. L'hélium permet au ballon de s'élever dans les airs car il le rend **tricoté**
The helium enables the ball to rise in the air because it makes it ***knitted*****.**
48. Pour ne pas la percuter, le véhicule ralentit quand il s’approche de la voiture immobile.
To avoid the collision, the vehicle slows down when it moves closer to the immobile car.

EMO
1. Quand j’ai eu le plus besoin d’elle, elle n’est même pas venue.
When I needed her the most, she did not even come. [sad]
2. Une heure de queue devant la poste, comme si j’avais du temps à perdre.
One hour queuing at the post office, as if I had time to loose. [angry]
3. J’ai finalement compris que je ne la reverrais plus.
I finally understood that I would never see her again. [sad]
4. J’ai été complètement perdue après notre rupture.
I was completely lost after our break-up. [sad]
5. Moi, j’ai eu tous mes partiels en juin.
I passed all my exams in June. [happy]
6. Ça a été super, mes vacances avec mon amie.
I had a great holiday with my friend. [happy]
7. Sa famille l’a abandonné, au cours de sa petite enfance.
His family abandoned him in his early childhood. [sad]
8. Nos vacances, elles sont vraiment formidables.
Our holiday is great. [happy]
9. Tu arrêtes de faire de l’œil à ma copine, abruti.
You, stop eyeing my girlfriend, idiot. [angry]
10. Ca suffit ces fermetures intempestives de la banque.
We have had enough of banks closing at inopportune times. [angry]
11. A ma grande joie, je vais prendre un verre avec un ami d’enfance.
To my delight, I am going to have a drink with a childhood friend. [happy]
12. Je vais réaliser mon rêve : partir en vacances dans les îles.
I am going to realize my dream: to go for a holiday in the Caribbean. [happy]
13. J’ai du mal à me contrôler devant cette nullité.
I am having trouble controlling myself in front of this crap. [angry]
14. Mes enfants, ils m’ont fait un super cadeau dimanche.
My children offered me a very nice present on Sunday. [happy]
15. Il y en avait marre de traîner ce boulet, je l’ai viré !
I was fed up with being burdened by him; I fired him! [angry]
16. Je suis heureux pour eux, ils s’aiment d’un amour rayonnant.
I am happy for them; their love for each other is radiant. [happy]
17. Depuis son accident, il ne voit plus personne.
Since his accident, he no longer sees anyone. [sad]
18. Tu as trouvé la maison de mes rêves à la campagne, merci beaucoup.
You found the house of my dreams in the countryside, thank you very much. [happy]
19. J’ai attendu pendant une heure dans le froid, c’est une honte.
I waited for one hour out in the cold; this is a shame. [angry]
20. Pendant des années, je me suis fait des illusions sur lui.
I have been deluding myself about him for years. [sad]
21. J’ai compté le peu de jours qu’il nous reste à vivre ensemble.
I have counted the days that we have left living together. [sad]

22. Une heure arrêtée en pleine campagne sans aucune explication, c'est intolérable !
A stop of one hour in the middle of the countryside without an explanation, that is unacceptable! [angry]
23. Ta décision, elle m'a révoltée au plus haut point.
Your decision outraged me at the highest level. [angry]
24. Elle me manque, ma famille, depuis que je suis ici.
Since I have been here, I miss my family. [sad]

25. Il a apporté un bouquet de fleurs sur la tombe de sa fille.
He brought a bunch of flowers on his daughter's tomb. [sad]
26. Il y a deux mois, il a été licencié, quel malheur.
Two months ago he got fired, what a misfortune. [sad]
27. Super, j'ai gagné beaucoup d'argent au loto.
Great, I won a lot of money at the lottery. [happy]
28. Je suis furieux, j'ai encore eu une prune aujourd'hui.
I am furious; I got a fine again today. [angry]
29. On a vainement tout essayé pour son cancer.
We tried everything in vain for his cancer. [sad]
30. Ce goinfre, il a avalé mes gâteaux préférés.
That greedy pig ate up my favourite cakes. [angry]
31. Tu n'as gardé qu'une unique lettre d'amour en souvenir d'elle.
You only kept one love letter as a memory of her. [sad]
32. J'ai encore retrouvé ma voiture neuve toute rayée, c'est inadmissible.
I found my new car scratched up again, this is unacceptable. [angry]
33. Tu vas laisser tous tes meilleurs souvenirs ici, si tu t'en vas.
If you go, you are going to leave all your good memories here. [sad]
34. Avec mes amies, j'ai vécu des moments inoubliables.
With my girlfriends, I have lived moments that I will never forget. [happy]
35. Je le coincerais un jour celui qui a saccagé ma maison.
I will catch him someday, the one who devastated my house. [angry]
36. Pour une bêtise, ma meilleure amie m'a quittée.
Because of a rubbish thing, my best friend left me. [sad]
37. Il a sauté au plafond en apprenant la nouvelle de ta nomination.
He jumped for joy on hearing that you were nominated. [happy]
38. Mon meilleur ami, il a remporté la médaille d'or au 100 mètres.
My best friend won the gold medal in the 100-meter race. [happy]
39. Je ne peux plus les supporter ces loubards.
I can't stand them anymore, those hooligans. [angry]
40. Demain, je vais me marier à Venise, le rêve.
Tomorrow, I will get married in Venice, what a dream. [happy]
41. J'ai la chance d'avoir des enfants merveilleux.
I'm lucky to have marvellous children. [happy]
42. C'est encore une grève des transports en commun, quelle bande de feignants.
Public transport is on strike again, what a bunch of slackers. [angry]
43. Espèce de peste, tu as encore fouillé dans ma chambre.
You pest, you searched my bedroom again. [angry]
44. Je suis tombé en extase devant ce tableau.
I felt ecstatic in front of this painting. [happy]
45. Malgré tous tes efforts, tu as échoué dans ton parcours professionnel.

Despite all your efforts, you failed in your career. [sad]
46. Regarde, il s'est encore garé devant la porte de notre garage.
Look, he parked in front of our garage door again. [angry]
47. Ce matin, on lui a annoncé la grave maladie de sa fille.
This morning, his daughter's serious illness was announced to him. [sad]
48. Je suis heureux à l'idée de m'installer avec toi.
I am happy at the idea of moving in with you. [happy]

PLAUEMO

1. Quand la poste a eu le plus besoin de facteurs, elle n'a même pas recruté
When the post-office needed more postmen, they did not even recruit.
2. Une minute de descente sur la piste, comme si j'avais du **chocolat** à vendre
*One minute sliding down the slope, as if I had **chocolate** to sell.*
3. On a finalement entendu que l'orage ne nous atteindrait pas
We finally heard that the storm would not reach us.
4. L'assiette a été complètement cassée après sa chute
The plate was completely broken after its fall.
5. A la mer, il y a du soleil tous les jours en juillet
By the sea, there is sun everyday in July.
6. Ca a été rapide, la chute des feuilles avec la tempête
That was quick, the leaves' fall with the storm.
7. Les cailloux l'ont bloquée, pendant sa descente
The pebbles stopped her, during her descent.
8. La chaudière, elle m'a réchauffé la grande maison rapidement
The boiler warmed up my large house quickly.
9. Il arrête de ranger des **buches** à l'intérieur, le poisson
*The fish stops tidying the **logs** inside.*
10. Ca tremble ces vieilles planches du ponton
They wobble, these old pontoon boards.
11. A la vieille ferme, les poules vont picorer les graines avec les autres volailles
In the old farm, the chickens will pick at the seeds with the other poultry.
12. Il va arrêter son moteur : glisser en roue libre dans la campagne
He will stop his engine: to slide freewheel in the countryside.
13. Le cerf-volant a du mal à s'élever dans l'air malgré le vent
The kite has difficulty arising despite the wind.
14. Les ananas, ils sont vraiment sucrés
The pineapples, they are really sweet.
15. Le remorqueur a beau jeu de trainer le paquebot, il l'a **maquillé**
*It is easy for the tugboat to drag the cruise ship; it put some **make-up** on it.*
16. La pente est douce pour la rivière, elle s'écoule d'une manière régulière
The slope is gentle for the river; it flows smoothly.
17. Depuis la cueillette, on ne trouve plus de **lampadaire**
*Since the harvest, one cannot find any **lamps**.*
18. Les **algues** ont trouvé un tracteur sur la roche de la berge, quel talent
*The **seaweeds** found a tractor under the riverbank's rock, what skill.*
19. La boule a tourné pendant une minute dans le flipper, c'est le **printemps**
*The ball turned during one minute in the pinball machine, it is **spring**.*
20. Pendant des années, elle s'est fait des tresses sur la tête
For years, she braided her head.
21. Elle a trié toutes les pages qu'il lui reste à imprimer en couleur

She sorted all the pages that she still has to print in colour.
22. Une heure exposé en plein soleil sans aucune protection : c'est risqué
One-hour sun exposure without protection, that is risky.
23. Son trajet, elle le lui a décrit pendant la pause
Her journey, she described it to him during the break.
24. Elle le nettoie, son frigo, depuis qu'elle est sous l'**eau**
*She cleans it, her fridge, since she is **underwater.***

25. Il a rapporté son jouet en plastique à l'entrée de sa niche
He brought back his plastic toy to the entrance of his kennel.
26. Il y a deux mois il a été rénové, quel **poids** !
*Two months ago it was renovated, what a **weight**!*
27. Bravo, elle a gagné beaucoup de voix aux élections
Congratulations, she won a lot of votes in the election.
28. Il est mûr, il a encore pris du soleil aujourd'hui
It is ripe; it took some sunlight again today.
29. On a vraiment tout ramassé pour les vendanges
We really collected everything for the grape-harvest.
30. Ce chameau, il a avalé des litres d'eau douce
This camel, he drank litres of freshwater.
31. Il n'a péché qu'une seule truite de rivière en souvenir des **casseroles**
*He fished only one river trout as a souvenir of the **saucepans**.*
32. On a encore retrouvé les remblais en béton submergés, c'est habituel
We found the concrete embankments flooded again, it is usual.
33. La crue va repousser toutes les grosses pierres sur le bord, si elle arrive
The tide will move away all the big stones on the bank, if it comes.
34. Avec les fleurs, le vase a fait un ensemble ravissant
With the flowers, the vase made a good-looking set.
35. Il la coincera sous la porte, la note qui signale le passage du facteur
He will wedge it under the door, the notice that signals the postman’s visit.
36. Pour une crevaison, le camion de livraison l'a **avalé**.
*For a flat tire, the delivery truck **ate** it.*
37. Le bouchon a sauté au plafond en évitant le lustre de la salle à manger
The cork jumped to the ceiling avoiding the ceiling light in the dining room.
38. Le meilleur vin, il a remporté la médaille d'or au **400 mètres**
*The best wine, it won the gold medal at the **400 meters**.*
39. Elle ne peut plus les porter ces chaussures
She cannot wear these shoes anymore.
40. Bientôt, les bourgeons vont s'ouvrir au soleil, c'est le printemps
Soon the buds will open under the sun; it is spring.
41. Il a l'avantage d'avoir des freins à disque
He has the advantage of having disk brakes.
42. C'est demain la saison des pluies en Asie, quel climat humide.
Tomorrow is the beginning of the rain season in Asia, what a humid climate.
43. Sorte de céréale l'avoine est encore cultivée dans les **autobus**
*Oat, a sort of cereal, is still cultivated in the **buses**.*
44. Les feuilles sont tombées en tas devant cette **date**
*The leaves fell down in heaps in front of this **date**.*
45. Malgré toutes les tempêtes, les tuiles sont restées dans **la boîte à gâteaux**
*Despite all the storms, the roof tiles stayed in the **cake box**.*

46. Regarde, le portail s'est encore ouvert devant l'entrée du square
Look, the gate has opened again in front of the park.
47. Ce matin, on lui a changé les vieux pneus de sa **télé**
*This morning, we changed the old tires of his **TV**.*
48. Il est glacé à l'idée de voyager avec la clim
He is freezing at the idea of travelling with the air conditioning.

GRAM
1. J'ai écouté la radio, étendu sur mon lit.
I listened to the radio, lying on my bed. [1]
2. Le voisin, il va tailler sa haie dans la matinée.
The neighbour is going to trim his hedge this morning. [3]
3. Tu as remonté l'horloge mécanique du salon.
You winded the mechanical clock in the living room. [2]
4. J'ai posé la valise sur le porte-bagages.
I put my suitcase on the luggage rack. [1]
5. Quand il est rentré, le repas était prêt.
When he came back, the meal was ready. [3]
6. Mon père, il va installer une échelle pour monter dans la grange.
My father is going to set up a ladder to go up in the barn. [3]
7. J'ai rangé mes vêtements dans le placard.
I put my clothes in the cupboard. [1]
8. Cet après-midi, j'ai ciré tous les meubles de la maison.
This afternoon I polished all the furniture in the house. [1]
9. Tu dois valider ton ticket dans le composteur.
You have to validate your ticket in the ticket-stamping machine. [2]
10. Ils ont fixé un panneau d'affichage dans la salle d'attente.
They put a notice board in the waiting room. [3]
11. Je vais mettre du carrelage dans ma salle de bain.
I will lay tiles in my bathroom. [1]
12. Les moutons, ils sont couchés dans la bergerie.
The sheep are lying in the sheepfold. [3]
13. Pour le goûter, j'ai préparé un gâteau au chocolat.
For a snack, I baked a chocolate cake. [1]
14. Tu as acheté une armoire ancienne à la brocante.
You bought an old cupboard at the second-hand shop. [2]
15. Pour gagner de l'argent, il a beaucoup travaillé.
To earn some money, he worked a lot. [3]
16. Le concierge, il a ramassé les papiers devant l'immeuble.
The caretaker collected the papers in front of the building. [3]
17. Le bus m'a déposée devant la gare.
The bus dropped me off in front of the station. [3]
18. Tu as repassé les vêtements dans la chambre.
You ironed the clothes in the bedroom. [2]
19. Quand il est parti, la lumière était éteinte.
When he left, the light was switched off. [3]
20. Le bateau de pêche file sur la rivière calme.
The fishing boat speeds off on the calm river. [3]
21. J'ai emmené les enfants à l'école aujourd'hui.
I took the children to school today. [1]

22. Après le travail, je me suis arrêté chez le boulanger.
After work, I stopped off at the baker's. [1]
23. Les couvertures, elles sont toutes dans le placard.
The blankets are all in the closet. [3]
24. J'ai feuilleté les nouvelles revues qui viennent d'arriver.
I flipped through the new magazines that just came out. [1]
25. Tu as tapé une lettre sur ton ordinateur portable.
You typed a letter on your laptop. [2]
26. Pour venir ce matin, j'ai pris l'autoroute
To come this morning, I took the highway. [1]
27. Il va ranger le vieux divan dans le grenier.
He will store the old sofa in the attic. [3]
28. J'ai battu les blancs en neige dans un saladier.
I beat the egg whites until stiff in a bowl. [1]
29. Les ouvriers, ils sont sur le chantier.
The workmen are at the construction site. [3]
30. Avant de bricoler la prise, j'ai coupé l'électricité.
Before installing the socket, I cut off the electricity. [3]
31. J'ai pris mon petit-déjeuner dans la cuisine, ce matin.
I had my breakfast in the kitchen, this morning. [1]
32. Mon bureau est situé dans ce bâtiment.
My office is located in this building. [3]
33. Quand tu es rentré, tu as allumé la télé.
When you came back, you turned on the TV. [2]
34. La semaine dernière, j'ai lavé la baignoire.
Last week, I cleaned the bathtub. [1]
35. Mon père, il a toujours une carte dans sa voiture.
My father always has a map in his car. [3]
36. J'ai porté son colis dans une boite aux lettres.
I put his parcel in a letterbox. [1]
37. Ma sœur, elle a rangé ses chaussettes de sport dans son sac.
My sister put away her sport socks in her bag. [3]
38. La table de la cuisine, elle est recouverte d'une toile cirée.
The kitchen table is covered with a tablecloth. [3]
39. J'ai changé mon bracelet de montre.
I changed my watchstrap. [1]
40. Avec ce vent glacial, il a mis un manteau pour sortir.
With this icy wind, he took a jacket to go out. [3]
41. J'ai attendu le feu vert avant de démarrer le jour de mon permis.
I waited for the green light before starting, the day of my driving licence. [1]
42. Ce matin, il a déposé le journal dans la boite aux lettres.
This morning, he put the newspaper in the letterbox. [3]
43. Tu as écrit la lettre sur du papier blanc.
You wrote the letter on white paper. [2]
44. Mon fils, il a rangé ses affaires d'école dans le bureau.
My son put away his school items in the desk. [3]
45. Avant de prendre l'avion, j'ai acheté les billets.
Before taking the plane, I bought the tickets. [1]
46. Tu as fait les lits avec des draps propres.
You made the bed with clean linens. [2]

47. Ce moineau, il va faire son nid dans le noisetier.
This sparrow is going to build his nest in the hazelnut tree. [3]
48. J’ai tourné la clé pour mettre le contact.
I turned the key to start the ignition. [1]
